# Biological clock function is linked to proactive and reactive personality types

**DOI:** 10.1186/s12915-018-0618-0

**Published:** 2018-12-21

**Authors:** Christian Tudorache, Hans Slabbekoorn, Yuri Robbers, Eline Hin, Johanna H. Meijer, Herman P. Spaink, Marcel J. M. Schaaf

**Affiliations:** 1 Institute of Biology, Leiden University, Leiden, The Netherlands; 2 Molecular Cell Biology, Leiden University Medical Center, Leiden, The Netherlands

**Keywords:** Circadian rhythm, Clock genes, Behaviour, Coping style, Endocrine regulation, Cortisol, Melatonin, RNA sequencing, Zebrafish

## Abstract

**Background:**

Many physiological processes in our body are controlled by the biological clock and show circadian rhythmicity. It is generally accepted that a robust rhythm is a prerequisite for optimal functioning and that a lack of rhythmicity can contribute to the pathogenesis of various diseases. Here, we tested in a heterogeneous laboratory zebrafish population whether and how variation in the rhythmicity of the biological clock is associated with the coping styles of individual animals, as assessed in a behavioural assay to reliably measure this along a continuum between proactive and reactive extremes.

**Results:**

Using RNA sequencing on brain samples, we demonstrated a prominent difference in the expression level of genes involved in the biological clock between proactive and reactive individuals. Subsequently, we tested whether this correlation between gene expression and coping style was due to a consistent change in the level of clock gene expression or to a phase shift or to altered amplitude of the circadian rhythm of gene expression. Our data show a remarkable individual variation in amplitude of the clock gene expression rhythms, which was also reflected in the fluctuating concentrations of melatonin and cortisol, and locomotor activity. This variation in rhythmicity showed a strong correlation with the coping style of the individual, ranging from robust rhythms with large amplitudes in proactive fish to a complete absence of rhythmicity in reactive fish. The rhythmicity of the proactive fish decreased when challenged with constant light conditions whereas the rhythmicity of reactive individuals was not altered.

**Conclusion:**

These results shed new light on the role of the biological clock by demonstrating that large variation in circadian rhythmicity of individuals may occur within populations. The observed correlation between coping style and circadian rhythmicity suggests that the level of rhythmicity forms an integral part of proactive or reactive coping styles.

**Electronic supplementary material:**

The online version of this article (10.1186/s12915-018-0618-0) contains supplementary material, which is available to authorized users.

## Background

In humans, personality comprises consistent individual variation of correlated behavioural and habitual traits [1], such as extraversiveness, impulsivity or novelty-seeking [2, 3]. These personality traits are also linked to the physiological and genetic makeup of an individual [4–8]. Similarly, in animals, behavioural traits such as risk-taking or aggressiveness [9] can be consistently correlated with physiological traits such as metabolic rate [10] or endocrine stress response [11]. These correlated sets of traits, termed coping styles (animal personalities), are recognised in a number of taxa [12] and vary along a proactive-reactive continuum, with proactive individuals being more risk-taker and aggressive and having higher baseline metabolic rates than reactive individuals [9, 10]. Further, characterisation of coping styles is crucial to a fundamental understanding of the occurrence of behavioural polymorphisms [13–15] and may provide new perspectives on the application of personalised care and personality disorders in humans [6, 16–21].

In experimental animals, coping styles can be assessed using the degree of risk-taking behaviour as a proxy, which is strongly correlated with a number of other behavioural and physiological traits [9–12]. In various fish species, risk-taking can be evaluated by an emergence test, a well-established test with high repeatability [9–11, 22]. During this test, a group of individual fish is allowed to emerge from a familiar shelter into a novel and potentially dangerous environment. The order of emergence is considered a measure for the individual tendency of risk-taking [9–11, 22].

In the present study, we have used this assay to assess the coping styles of individual zebrafish from a heterogeneous laboratory population (AB/TL strain). Subsequently, RNA sequencing was performed on brain samples from proactive and reactive zebrafish to further investigate molecular determinants of coping styles. Our results showed an overrepresentation of genes involved in the biological clock among the genes differentially expressed in the brains from proactive and reactive individuals. The biological clock is an endogenous timing mechanism which allows an organism to anticipate regular changes in the environment that result from the day/night cycle [23–27]. In vertebrates, the molecular basis of the biological clock is formed by feedback loops of gene expression. In the core loop, the transcription factors BMAL and CLOCK stimulate the expression of the genes encoding the corepressor proteins PER and CRY, which inhibit the activity of BMAL and CLOCK. Thereby, PER and CRY repress their own expression and create an oscillating pattern of gene expression. This oscillator is stabilised by a second feedback loop which involves the nuclear receptors REV-ERB and ROR. In addition, the biological clock can be synchronised with the actual day-night cycle by environmental cues like light and temperature. The suprachiasmatic nucleus (SCN) of the hypothalamus in mammals and the pineal gland in other vertebrates appear to act as ‘master clocks’. They coordinate the clock of peripheral cells and tissues through a set of systemic signals, including the cycling secretion patterns of the hormones melatonin and cortisol. This way, the biological clock creates diurnal rhythmicity which can be observed at several functional levels, from the oscillating expression of clock genes over endocrine secretion patterns to rhythmic behavioural activity [23–27].

The association between clock gene expression levels and coping styles observed in our RNAseq experiments led to three alternative hypotheses. The different gene expression levels may be generally higher or lower between individuals with different coping styles; the observed differences in gene expression may be due to a shift in phase of the biological clock (i.e. chronotype [4, 23–25, 28]) or to a different amplitude of the diurnal rhythm. We tested these hypotheses on three levels of biological function: on the molecular level by measuring gene expression, on the endocrine level by investigating hormone production and on the behavioural level by studying locomotion activity. Our results show that the amplitude of the circadian rhythm correlates with the coping style of the individual, ranging between high amplitudes in proactive fish and an absence of rhythmicity in reactive fish.

## Results

### The emergence test as a proxy for coping style

**Fig. 1:**
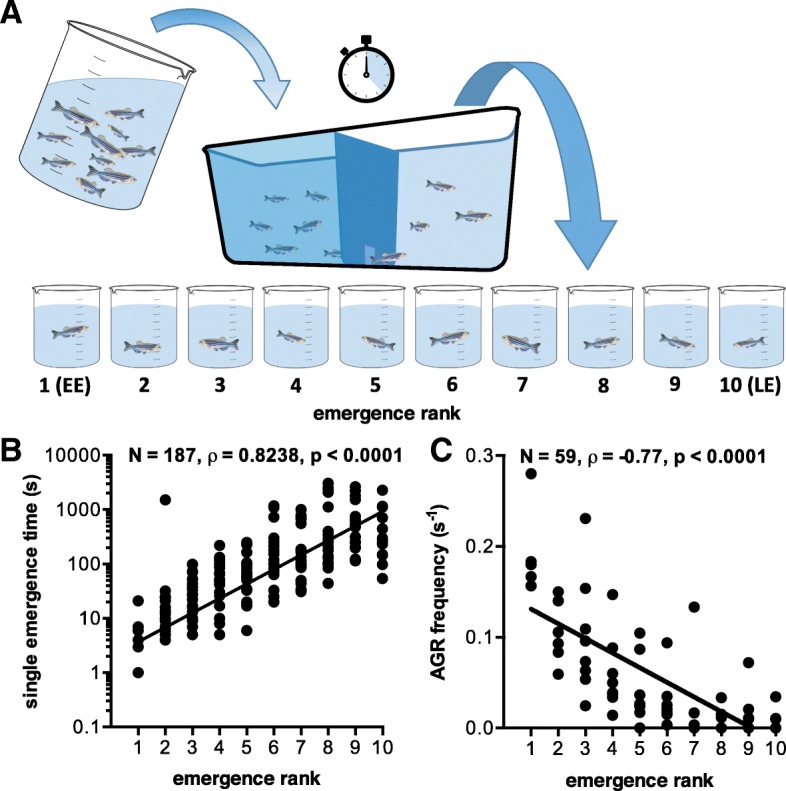
Behavioural testing in zebrafish. Behavioural testing in zebrafish. **a** Schematic overview of the group emergence test used in this study. A fish tank was used that consisted of two compartments separated by a wall with a closable hatch. A batch of ten fish was introduced into the darkened holding compartment of this tank. After a 10-min acclimation period, the hatch was opened and fish were allowed to emerge into the uncovered, well lit, second compartment, and the emergence time was recorded. After emergence, individual fish were collected and grouped according to emergence rank (1–10), with number 1 designated early emerger (EE) and number 10 late emerger (LE). The emergence order is considered a measure for risk-taking behaviour, which is a widely used proxy for coping style. **b** Behavioural traits are correlated across situation and time: single emergence time plotted against group emergence ranks. Data were collected from 24 batches of 10 adult zebrafish (*N* = 187) in the group emergence test (rank) and subsequent single emergence test (time), with a significant correlation between emergence rank in the group test and time in the single test (Spearman rank test). **c** Aggressiveness correlates with risk-taking behaviour within a coping style: individuals previously ranked during a group emergence test were subjected to a mirror-image stimulation. Upon exposure to their own mirror image, the number of aggressive behaviours (AGR; bites to the image, parallel swimming, circles and strikes) were counted and divided by the measuring period minus the duration of freezing bouts and approach latency. The resulting AGR frequency (s^−1^) was significantly correlated with emergence rank (Spearman rank test, *N* = 59)

We assessed different coping styles using a group emergence test, in which each zebrafish of a group of ten was allowed to emerge from a familiar (slightly darkened) shelter into a novel and potentially dangerous environment. The individual fish were ranked according to their latency to emerge (Fig. 1a). We evaluated the temporal consistency of this emergence test and tested whether difference in light intensity was a confounding factor. First, the test was repeated in a single emergence setting and emergence times were measured. A positive correlation between single emergence times and group emergence rank was observed (Fig. 1b; Spearman rank, *N* = 187, *ρ* = 0.82, *p* < 0.0001). Second, single emergence tests were performed on two consecutive days at different times of the day. The results of this experiment show strong correlations between single emergence times of the same individual during two consecutive days at different times of the day (Additional file 1: Figure S1A; Spearman rank, *N* = 19–24, *ρ* = 0.38–0.91, *p* < 0.001 or *p* < 0.0001). Third, to test the effect of light conditions on emergence behaviour, the emergence test was performed with similar light conditions in both the shelter and the novel environment compartment. A strong correlation was observed between the emergence time measured using this approach and the emergence rank in the initial test (Additional file 1: Figure S1B; Spearman rank, *N* = 72, *ρ* = 0.77, *p* < 0.0001). Fourth, when plotting all pooled emergence times from single emergence experiments over the group emergence times, a strong positive correlation confirmed the robustness of emergence time regardless the social and environmental settings (Additional file 1: Figure S1C; Spearman rank, *N* = 190, *ρ* = 0.87, *p* < 0.0001). In order to further evaluate risk-taking as a proxy for coping style, we tested and confirmed a correlation between emergence rank and frequency of aggressive behaviour during a mirror-biting test (Fig. 1c; Spearman rank, *N* = 59, *ρ* = − 0.77, *p* < 0.0001).

Since fish from the hybrid AB/TL line were used in these experiments, it could be hypothesised that the observed behavioural variation in this line originated from genetic differences between the original AB and TL lines. Since these two lines have previously been shown to perform differently in other behavioural tests [29], proactivity may have been inherited from one line and reactivity from the other. However, no difference was observed in the behaviour of fish from the AB and TL lines in the emergence test (Additional file 2: Figure S2; *X*^2^ test, *N* = 6, *p* > 0.05). We therefore conclude that genetic variation in the hybrid AB/TL line which may underlie the observed phenotypical variation most likely originates from genetic variation within the original AB and TL strains, [30, 31]. However, the contribution of genetic variation between these lines (e.g. involving different genes affecting the phenotype in the two lines) can still not be excluded [30, 32].

### Diurnal rhythmicity of gene expression is associated with coping style

**Fig. 2:**
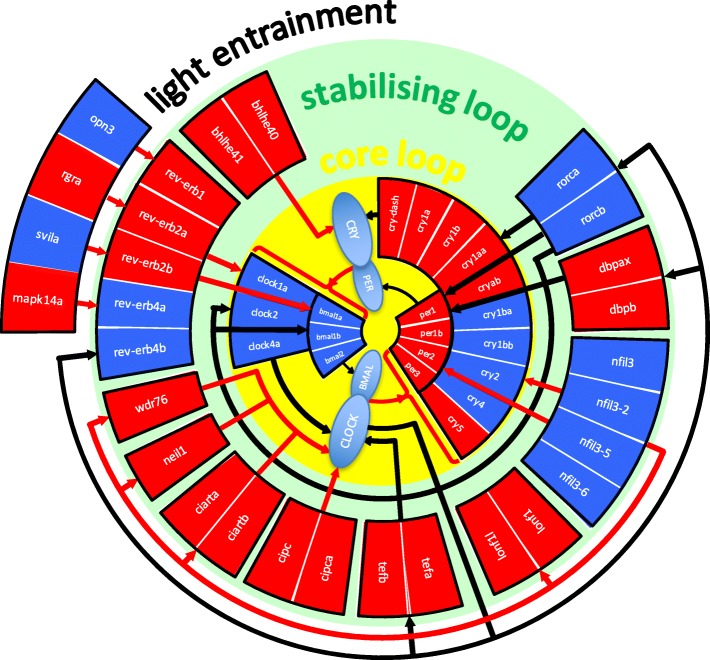
Transcriptome analysis of brain samples from proactive (early emerging, EE) and reactive (late emerging, LE) zebrafish using RNA sequencing. Brains were collected from EE and LE fish (*n* = 4) at 7 hCT, and subsequently, RNA was isolated and used for RNA sequencing. Analysis of the EE and LE transcriptomes showed that differences in gene expression were found for 3% of genes involved in the regulation of the biological clock (see Additional file 3: Table S1). This overview shows differential gene expression levels of 44 differently (*p* < 0.05) regulated genes, involved in the core loop, the stabilising loop and the entrainment of the biological clock. Rectangles represent genes, and differential gene expression between EE and LE fish is indicated by red (higher expression level in EE) and blue rectangles (higher level in LE fish). Circles represent regulating protein complexes. Inhibition is indicated by a red line with round head, and promotion by a black line with arrow head

Subsequently, group emergence tests were performed with ten individuals and we collected individuals of rank 1 (early emergers, EE, highly risk-taking) and rank 10 (late emergers, LE, highly risk-avoiding). RNA was isolated from their brains and processed for transcriptome analysis by RNA sequencing. This yielded RNA sequences derived from 31,398 different genes. The expression of 1478 of these genes differed significantly between EE and LE fish (Additional file 3: Table S1). Gene ontology analysis revealed that 20 of the top 100 most differentially expressed genes were involved in the control of the biological clock (and 43 of the total of 1478 differentially expressed genes, enrichment score 3.66; Fig. 2 and Additional file 3: Table S1), with a fold change ranging from 4.34 to 20.07. These data indicate a strong association between coping styles and the regulation of the biological clock. Other enriched ontology groups to be found in the top 100 most differentially expressed genes were ‘oxygen binding and transport’ (enrichment score 5.81), ‘transcription factor activity’ (3.38) and ‘haematopoiesis’ (3.30).

**Fig. 3:**
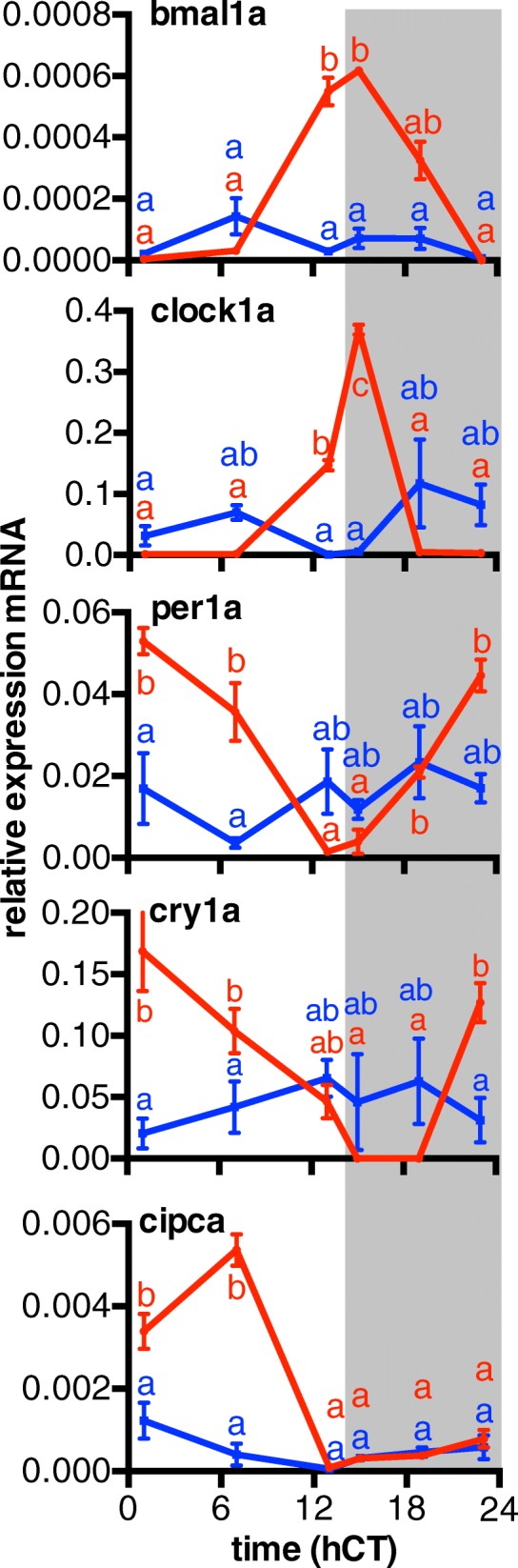
The expression patterns of five clock-related genes over time are correlated with the risk-taking behaviour. The brains were collected from EE and LE fish, at six different time points, i.e. 1, 7, 13, 15, 19 and 23 hCT, in a 14–10-h light to dark regime (*n* = 3), and subsequently, RNA was isolated and used for qPCR analysis. The relative expression of *bmal1a*, *clock1a*, *per1a*, *cry1a* and *cipca* was double-plotted over time from EE (red) and LE (blue) fish. Letters indicate significant difference (two-way ANOVA, with time and coping style as variables, Bonferroni post hoc test, *p* < 0.05, *N* = 6. Data shown are means ± SEM)

Subsequently, we further investigated the relationship between coping style and the biological clock at different functional levels, i.e. gene expression, endocrinology and locomotion behaviour. First, we measured, by quantitative PCR, the expression patterns of 5 genes, i.e. one member from each of the four core clock genes and one stabilising loop gene that showed diurnal rhythmicity in its expression (*bmal1a*, *clock1a*, *per1a*, *cry1a* and *cipca*) and that were among the top 20 most differentially expressed clock genes. The observed expression patterns show that EE fish had higher peak values and a wider range of expression levels of these genes than LE fish (Fig. 3; Two-way ANOVA, Bonferroni post hoc, *N* = 6, *p* < 0.05). When fish of all emergence ranks were taken into account (i.e. 1 to 10, Additional file 4: Figure S3), both the area under the curve (AUC, as a measure for net transcriptional activity) and the amplitude of expression (used as an indicator for rhythmicity) were negatively correlated with the emergence rank, for *bmal1a* and *cipca* regarding AUC (Spearman rank test, *N* = 72, *ρ* = − 0.4619, *p* = 0.0102 and *ρ* = − 0.4793, *p* = 0.0074, respectively) and all genes regarding amplitude (Spearman rank test, *N* = 72, *ρ* = − 0.67 to − 0.45, *p* < 0.0001 to *p* = 0.0138). These results indicate a more robust circadian rhythmicity in individuals with proactive coping styles compared to individuals with reactive coping styles.

### Diurnal rhythmicity of hormone secretion is associated with coping style

**Fig. 4:**
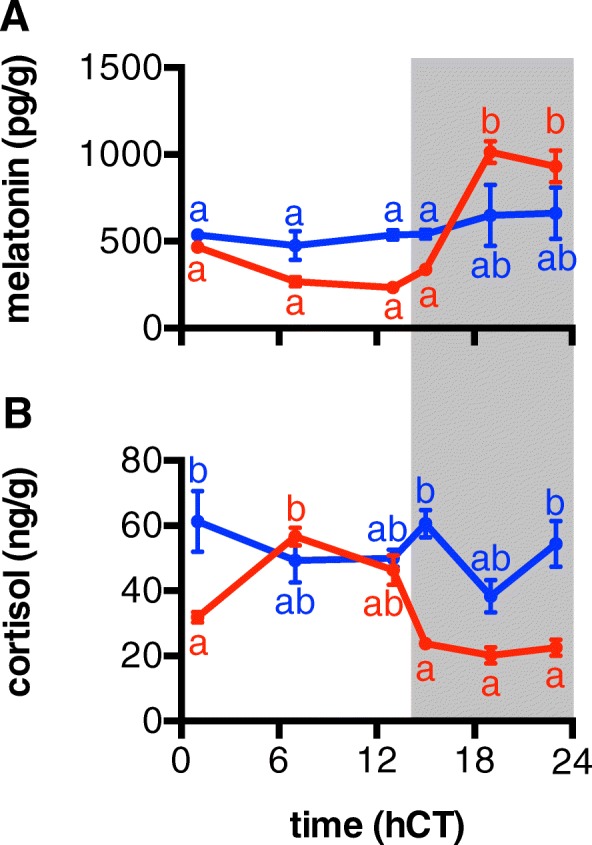
Diurnal patterns in cortisol and melatonin levels are associated with the risk-taking behaviour. Full body melatonin (**a**) and cortisol (**b**) concentrations (pg g^−1^ and ng g^−1^ body weight, respectively) were determined by ELISA in early (EE, red) and late emerging fish (LE, blue) over 24 h during a 14:10 light to dark cycle (indicated by white and grey bars, double plotted). Samples were taken at 1, 7, 13, 15, 19 and 23 h circadian time (hCT). Data shown are means ± SE (*N* = 3). Letters indicate significant difference (two-way ANOVA, with time and coping style as variables, Bonferroni post hoc test, *p* < 0.05, *N* = 6. Data shown are means ± SEM)

Subsequently, we studied the diurnal rhythmicity of levels of cortisol and melatonin, important endocrine regulators of diurnal activity patterns [23]. In EE fish, whole body melatonin and cortisol concentrations fluctuated significantly between light and dark phase, whereas LE fish had constantly high values for cortisol and intermediate values for melatonin, with no detectable rhythmicity (Fig. 4; two-way ANOVA, with time and coping style as variables, Bonferroni post hoc test, *N* = 6, *p* < 0.05). Additionally, taking the data of all emergence ranks into account, a positive correlation between emergence rank and net secretion activity (AUC), and a negative correlation between emergence rank and circadian rhythmicity (amplitude), was observed for both hormones (Additional file 5: Figure S4; Spearman rank test, *N* = 72; cortisol: *ρ* = 0.81 and *p* < 0.0001 (AUC), *ρ* = − 0.64 and *p* = 0.0002 (amplitude); melatonin: *ρ* = 0.61 and *p* = 0.0003 (AUC), *ρ* = − 0.37 and *p* = 0.0429 (amplitude)). This indicates a stronger rhythmicity in the secretion of melatonin and cortisol in proactive individuals compared to reactive individuals.

### Diurnal rhythmicity of behavioural activity is associated with coping style

**Fig. 5:**
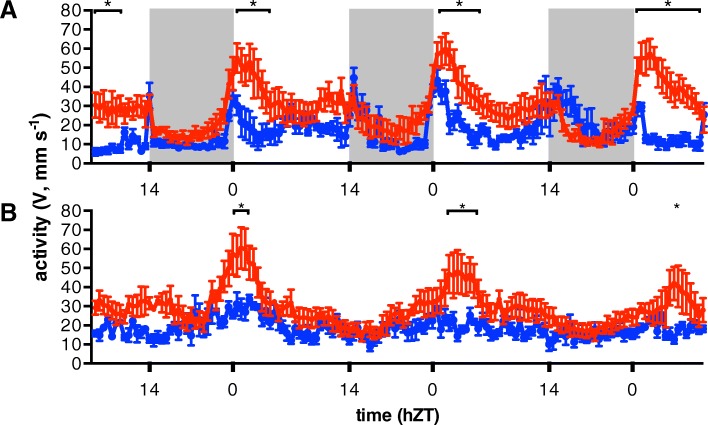
Diurnal activity pattern is associated with emergence behaviour. Actogram of early (EE, emergence rank 1, red) and late emerging fish (LE, rank 10, blue) during 3 days under light-dark regime (**a**) and during three subsequent days in constant light (**b**). Locomotion activity is represented as average swimming velocities over 30 min (*V*, mm s^−1^) as a function of time (hours circadian time, hCT). Activity recording started at 7 hCT of day 1 and ended at 7 hCT of day 6. The light regime switched at day 3 from light-dark (14:10) cycles to constant light, indicated by white and grey bars. Asterisks and horizontal bars indicate significant different time intervals between coping styles (two-way ANOVA with time and coping style as variables, Sidack’s post hoc test, *N* = 6, *p* < 0.05. Data shown are means ± SEM)

Finally, we monitored the locomotor activity during three consecutive diurnal cycles. Plotting average swimming velocity data of EE and LE fish revealed that EE fish reached higher peak activity levels (especially in the first hours of the light phase) and a wider range of activity levels than LE fish (Fig. 5a; two-way ANOVA with time and coping style as variables, Sidack’s post hoc test, *N* = 6, *p* < 0.05). Further analysis (Spearman rank) of the activity data using fish of all emergence ranks showed that emergence rank was negatively correlated with rhythmicity (Additional file 6: Figure S5A, *N* = 72, *ρ* = − 0.34 and *p* < 0.0037 (AUC)), total behavioural activity (Additional file 6: Figure S5B, *N* = 72, *ρ* = − 0.39 and *p* < 0.0008 (amplitude)) and rhythm strength (Additional file 6: Figure S5C, *N* = 72, *ρ* = − 0.53 and *p* < 0.0001). However, maximum swimming speeds did not vary significantly with emergence ranks, suggesting that there was no difference in physiological capacity for locomotion between fish of different emergence ranks (Additional file 6: Figure S5D). Again, these data demonstrate a more pronounced rhythmicity in the more proactive individuals.

Finally, we subjected the fish to a constant light challenge and monitored their behaviour over three consecutive diurnal cycles. Under these constant light conditions, the differences between EE and LE fish in maximum values and range of activity level gradually decreased, with EE fish approaching the activity pattern observed for LE fish (Fig. 5b; two-way ANOVA with time and coping style as variables, Sidack’s post hoc test, *N* = 6, *p* < 0.05). The activity data of fish of all emergence ranks showed that rhythm strength was correlated (Spearman rank) with emergence rank (Additional file 6: Figure S5G; *N* = 72, *ρ* = − 0.61, *p* < 0.0001), but not total activity or amplitude (Additional file 6: Figure S5E-F, *N* = 72, *p* > 0.05). These results indicate that activity patterns of proactive individuals gradually lose rhythmicity in the absence of time-related cues and become similar to the arrhythmic behaviour of reactive individuals. This demonstrates that the strong diurnal rhythmicity of proactive individuals is dependent on external light cues.

## Discussion

In the present study, we have classified individual zebrafish from the same batch by their coping style, along a proactive-reactive continuum, using risk-taking behaviour as a proxy for coping style. Subsequently, we have demonstrated a strong association between coping style and the amplitude of the circadian rhythmicity at the molecular, endocrine and behavioural level: proactive individuals showed a robust diurnal rhythm with a large amplitude, whereas reactive individuals virtually lacked rhythmicity.

Transcriptome analysis using RNA sequencing on brain samples from proactive and reactive individuals showed an enrichment of genes involved in the regulation of the biological clock among the set of genes that was differentially expressed. In several recent studies, transcriptome analysis has also been performed to study differential gene expression related to coping styles in zebrafish [9, 33]. In one of these studies [9], an approach to assess coping styles was used which was very similar to ours, and the RNA sequencing analysis on hindbrain samples demonstrated that genes involved in the biological clock were overrepresented among the differentially expressed genes. However, in other brain regions, no correlation between clock-related genes and coping style was observed. In another study [34], a microarray analysis was performed on whole brain samples from zebrafish strains with different behavioural phenotypes, which also revealed differential expression of biological clock-related genes.

The results from our RNA sequencing raised two issues, which we answered in additional control experiments. First, the time of the day may have affected the separation of behavioural phenotypes during the emergence test, and the observed differences in coping style could have been a result of phase-shifted diurnal rhythmicities (i.e. chronotypes [4, 23–25, 28]). However, the performance in the emergence test of individual fish at different times on two consecutive days appeared to be highly consistent, indicating that the observed behaviour in the emergence test was independent of the time of the day. Second, many of the differentially expressed clock genes are known to be light-sensitive [35]. Therefore, the issue arose whether light responsiveness was a confounding factor in the emergence test, since in the initial setup there was a difference in light intensity between the two compartments. When the test was repeated with identical light conditions on both sides of the hatch, the emergence order was not altered, which demonstrated that the emergence order was not affected by differences in light responsiveness between individual fish.

In addition, we tested the validity of risk-taking behaviour in the emergence test as a proxy for coping style by correlating it with aggressiveness measured using a mirror test. We found a strong correlation between these two behavioural traits, which is in line with previous studies using an evolutionary model [13] and an experimental approach [36] and has been reviewed elaborately [14, 37, 38]. The results of our extensive validation of the emergence test support the robustness of the emergence test and explain its wide use in coping style research [9–11, 22].

In our population of zebrafish, we found large individual variation in diurnal rhythmicity within a continuum that ranges from very robust diurnal rhythms to a complete absence of rhythmicity. The spontaneous occurrence of individuals with a low level of rhythmicity within a healthy laboratory population has been shown before in Djungarian hamsters [39], and it will be interesting to study whether such individuals can be found under natural conditions as well. Under certain conditions, a lack of an internal rhythmicity can be advantageous for individuals, for example when external cues are absent or fluctuate unpredictably [23–25, 28] or when competition with other individuals can be avoided by a larger flexibility in the timing of certain activities [40]. In humans, the enormous variation observed in the level and rhythm of the melatonin secretion [41–43] suggests that variability in circadian rhythmicity may be a more general phenomenon than previously assumed. Recognition of this diversity in individual phenotypes [12–15] will be of great relevance for our understanding of the regulation and function of the biological clock, but also for the personalization of diagnosis and treatment of psychiatric and metabolic disorders [23–25, 28, 44] and degenerative diseases [45] that are attributed to dysregulation of the biological clock.

The observed variation of circadian rhythmicity was associated with coping style, with proactive individuals showing robust rhythms and reactive fish displaying very low rhythmicity. We therefore suggest the diurnal rhythmicity to be an integral part of the coping style of an individual. In general, proactive individuals have been shown to be mainly intrinsically organised, i.e. routine-based and relatively resistant to the influence of external stimuli [12–14]. The biological clock itself is a proactive system, anticipating rhythmic changes in external conditions, so the stronger rhythmicity found in proactive zebrafish is in line with a more intrinsic regulation of diurnal rhythmicity. In contrast, for reactive individuals, a robust diurnal rhythm may be constraining the flexibility that characterises their reactive phenotype and would therefore be maladaptive in combination with this behavioural phenotype.

Although future research should indicate how generally this phenomenon occurs in nature, our study demonstrates that there are mechanisms that under certain circumstances link coping style and circadian rhythmicity. Possibly, this correlation between coping style and circadian rhythmicity may be a result of genetic correlation, i.e. one trait responding as a consequence of selection acting upon another trait with which it shares genes [46]. The main mechanism underlying genetic correlation is pleiotropy, in which one genetic variant affects more than one phenotype [47]. Genes related to the biological clock are often pleiotropic genes, which can influence both behaviour and physiology [23–25, 27, 28, 48]. Other possible pleiotropic genes that are involved in the observed correlation between coping style and the biological clock are those involved in the biosynthesis and function of neurotransmitters and hormones. For example, cortisol has been found to be a ‘permissive cue’ for diurnal rhythmicity through a variety of molecular mechanism [49]. Additionally, glucocorticoids are responsible for many changes in behaviour related to coping style in both humans [50] and non-human vertebrates [51]. Besides this direct form of pleiotropy, an indirect form of pleiotropy exists, in which a genetic variant affects one phenotype, which lies on the causal path to a second phenotype. In our model, changes in circadian activity patterns may have dramatic effects on certain endocrine systems [52, 53], which in turn may influence other behavioural traits.

## Conclusions

It is generally accepted that a disturbed or weak diurnal rhythm compromises optimal biological functioning and is linked to psychiatric and metabolic disorders and degenerative diseases. However, here, we demonstrate large variation in diurnal rhythmicity within a single laboratory population of zebrafish, ranging from robust rhythms to a complete absence of rhythmicity. This variation is correlated with the coping style of the individual fish. This association suggests that the biological clock may be part of the complex phenotypes that represent individual survival strategies in nature as well.

## Materials and methods

### Zebrafish husbandry conditions

Zebrafish were maintained and handled according to the guidelines from the Zebrafish Model Organism Database (ZFIN, http://zfin.org) and in compliance with the directives of the local animal welfare committee of Leiden University (DEC number 14058). The wildtype zebrafish (*Danio rerio*) strain used in this study was an AB/TL strain, which originates from crossbreeding of the AB and Tüpfel Long Fin (TL) strains. This strain was originally obtained from the Hubrecht Laboratory (Utrecht, The Netherlands) and maintained in our laboratory for at least ten generations at the time of the experiments described here. New generations were generated by mating of fish from the previous generation, using 60–80 individuals (1:1 ratio male to female). The AB/TL line, considered a segregated hybrid line, was chosen because it contains large genetic variation. In addition to the genetic variation existing within the original AB and TL strains [30, 31], the variation between the two original lines [30, 32] also contributes to the genetic variation of the AB/TL line.

The fish were reared in densities of ± 40 individuals (male to female 1:1) per 7.5-l tanks in standardised recirculation systems (Fleuren & Nooijen, Nederweert, The Netherlands); water temperature was maintained at 28 ± 1 °C (*n* = 5), with a conductivity of 518 ± 12 μS (*n* = 5) and oxygen concentration of 7.9 ± 0.4 mg l^−1^ (*n* = 5). Light cycles were maintained at 14-h light to 10-h darkness cycle, with light periods from 8:00/7:00 (0 h Circadian Time, (hCT)) to 22:00/21:00 (14 hCT) summer time/winter time, with a linearly decreasing/increasing light intensity between 0 and 320 ± 21 ln m^−2^ (*N* = 3) over a period of 15 min. Fish were fed twice daily, at 1 ± 1 hCT and at 8 ± 1 hCT, with dry food (DuplaRinM, Gelsdorf, Germany) and frozen artemia (Dutch Select Food, Aquadistri BV, Klundert, The Netherlands). The fish used in the experiments were between 1 and 2 years old and had a standard length of 32.1 ± 2.3 mm and a body weight of 150.61 ± 17.99 mg (mean ± SD). There was no correlation between emergence rank and body weight (Spearman rank, *N* = 144, *p* > 0.05).

### Behavioural tests

#### Group emergence

The setup for the emergence test consisted of a Plexiglas tank (33 × 13 × 13 cm) with a volume of 3 l. It was divided into a darkened holding compartment and an uncovered novel area compartment, by a wall with a hatch (2 × 2 cm) at its mid bottom. The hatch was manually closable by means of a trap door. Ten fish of mixed sex were transferred from the housing tank into the holding compartment of the emergence setup where they were acclimated for 10 min. Thereafter, the trap door was opened, enabling the emergence of one fish at a time into the novel area compartment. After each emergence event, the trap door was closed and the emerged fish was manually transferred to holding tanks (33 × 13 × 13 cm), separated by emergence rank (1–10). The time of emergence from the moment of the opening of the trap door until the actual passage through the hatch was recorded for all individuals. The entire test did not last longer than 10 min and was performed between 5:00 to 8:00 hCT, unless otherwise indicated. Fish not emerging after 10 min were excluded from further analysis. Animals were kept overnight in the holding tanks until further experimentation the next day.

#### Individual emergence

In order to test the consistency of the degree of risk-taking over the diurnal cycle, individual emergence was investigated at three times of the day (1:00 h, 7:00 h and 13:00 hCT). Nine groups (*n* = 8) were created, each tested at a combination of two different times of the day on consecutive days (1:00/1:00, 1:00/7:00, 1:00/13:00, 7:00/1:00, 7:00/7:00, 7:00/13:00, 13:00/1:00, 13:00/7:00, 13:00/13:00 hCT). The individual emergence setup consisted of three vertical rows of eight Plexiglass emergence tanks, as used for the group emergence tests, with the trap doors connected per row. A flow through system (47.6 l h^−1^ per tank) provided each tank with fresh system water, and LED strips (Eurolite, Steinigke Showtechnic GmbH, Waldbüttelbrunn, Germany) were placed in front of the uncovered compartment, resulting in similar light intensity as in the husbandry facility (425 ± 11 ln m^−2^). The light intensity of the holding compartment was 5 ± 1 ln m^−2^. This setup enabled testing the emergence of eight individuals simultaneously. Fish were placed individually in the holding compartment and acclimated overnight. At testing time, hatches were opened manually and emergence was recorded using a video camera (HDC-SD90, Panansonic Corporation, Kadoma Osaka, Japan) placed 1.2 m in front of the setup. The test was terminated when all fish had emerged. Fish were placed back into the holding compartment, and the test was repeated on the same individual the following day, according to the design described above.

In order to test the consistency of risk-taking behaviour at identical light intensities of the holding compartment and the novel environment compartment of the emergence test, a similar setup was used with light intensities of 5 ± 1 ln m^−2^ in both compartments. Here too, nine groups with eight fish each (*N* = 72) were created, each tested at 7:00 h ZT in the individual emergence setup according to the protocol described above.

A group emergence test was performed 1 day prior to the individual emergence tests to investigate the correlation between the behaviour in the two tests. Each row of 8 tanks in the individual emergence setup contained fish from the same group emergence test. Since only 8 out of the 10 individuals could be tested simultaneously, two individuals were randomly eliminated, using the excel randomisation function. The group emergence test was performed nine times, yielding 72 fish (9 groups of 8) to be tested in the individual emergence test. As indicated above, each group was tested at different times on two consecutive days. This combination of individual and group emergence test was performed three times, so a total of 216 fish was tested. Individual emergence times were determined by the observation from the video footage and were correlated with the emergence time of the group emergence test using Spearman rank correlation with significance accepted at *p* < 0.05.

#### Emergence of mixed AB and TL groups

In order to test whether the observed behavioural variation in the degree of risk-taking originated from the difference between the AB and TL lines used to create the wildtype (AB/TL) line of this study, the emergence test was repeated with groups created by mixing AB and TL fish in a ratio of 1:1. Six groups of ten fish were created before the group emergence test was performed, and housed under the same conditions as previously described. The group emergence test was subsequently performed for each group according to the protocol described above. Data analysis was performed using a *Χ*^2^ test (*N* = 6, significance accepted at *p* < 0.05).

#### Mirror-image stimulation

For the mirror-image stimulation (15), three rows of eight Plexiglas tanks (33 × 13 × 13 cm, capacity 3 l) were placed on a white table surface (1.0 × 1.0 m). A HD video camera (Panasonic, HDC-SD90, Panasonic Inc., Japan) was mounted 1.7 m above the table. Fish previously tested in the group emergence test (ca. 2 hCT) in sets of ten (with random elimination of two) were placed individually in a circular holding chamber, consisting of a grey tube (10 cm diameter, 15 cm height) placed vertically in the middle of the tanks. A vertical mirror (13 × 15 cm) was placed at one of the short ends of the tank. Each row contained fish from the same group emergence test, i.e. 8 fish in three rows, resulting in 24 fish per experimental run, with three experimental runs. Fish were acclimated 30 min prior to recording, after which the holding chambers were removed simultaneously from all 24 tanks in a swift vertical movement, exposing them to the open field and the mirror, and their behaviour was recorded for 10 min (ca. 4 hCT). Measured variables were the duration of freezing bouts (FRZ; complete lack of movement only for eyes and gills), the latency to realise the first approach to the mirror until a distance of 1 body length, i.e. ca. 4 cm, i.e. 1 body length (LFA), and the number of aggressive behaviours (AGR; bites to the image, parallel swimming, circles and strikes). The variables were determined by the observation from the video footage. During subsequent analysis, AGR were counted and divided by the measuring period minus the FRZ and LFA, therefore regarding only the period dedicated to potentially aggressive interactions with the mirror image. Non-responsive fish, i.e. fish with FRZ of 10 min or longer, were excluded from the analysis. The correlation between the resulting AGR frequency (s^−1^) and the emergence time of the group emergence test was evaluated using Spearman rank correlation (*n* = 59, *N* = 9, significance accepted at *p* < 0.05).

### Transcriptional analysis

#### RNA sequencing

In order to establish a first estimation of the range of RNA expression levels over emergence rank variation, four individuals of emergence rank 1 (EE) and four individuals of emergence rank 10 (LE) were sacrificed in ice water, at 7 ± 0.5 hCT. Their brains were dissected out and preserved in RNALater (Ambion, Austin, TX). Brains were homogenised in 500 μl of Trizol reagent (Qiagen). Total RNA was extracted and column-purified using the RNeasy MinElute Cleanup Kit (Qiagen), according to the manufacturer’s instructions. RNA samples were treated with DNaseI (Life Technologies) to remove residual genomic DNA. RNA integrity was analysed by Lab-on-a-chip analysis (Agilent, Amstelveen, The Netherlands). A total of 2 μg of RNA was used to make RNAseq libraries using the Illumina TruSeq RNA Sample Preparation Kit v2 (Illumina, Inc., San Diego, CA, USA) according to the manufacturer’s instructions (with two minor modifications: in the adapter ligation step, 1 μl, instead of 2.5 μl, adaptor was used, and in the library size selection step, the library fragments were isolated with a double Ampure XP purification with a 0.7× beads to library ratio (Beckman Coulter, Woerden, The Netherlands)). The resulting mRNAseq library was sequenced using an Illumina HiSeq2500 Instrument (Illumina, Inc.) with a read length of 2 × 50 nucleotides. Image analysis and base-calling were done using the Illumina HCS version 2.0.12. The raw data has been submitted to the GEO database (accession number GSE64570). The data was analysed using the GeneTiles software (http://www.genetiles.com) with a cutoff *p* value of 0.05. In brief, Genetiles used fastq files as input for the program Bowtie2 (http://bowtie-bio.sourceforge.net) to align the reads to the zebrafish genome (obtained from Ensembl version Zv9). Subsequently, the programs SAMtools (http://samtools.sourceforge.net), DESeq2 and DESeq (http://bioconductor.org) were used for data processing. Gene ontology of the significantly (*p* < 0.05) different regulated 1478 genes was analysed using the online functional classification tool DAVID (http://david.abcc.ncifcrf.gov/summary.jsp). In addition, for genes not classified by DAVID, information was gathered on their function, using the websites GeneCards (http://www.genecards.org/), NCBI (http://www.ncbi.nlm.nih.gov/gene) and Genetics Home Reference (http://www.ncbi.nlm.nih.gov/gene). Using this information, all genes involved in the biological clock were identified. In the “Results” section, we mention the enrichment scores of the three most enriched functional annotation clusters for the top hundred most differentially expressed genes, assigned by DAVID.

#### Quantitative PCR

For the estimation of transcriptomic regulation, one member from each core clock gene family was selected (*bmal1a*, *clock1a*, *per1a*, *cry1a*), and one gene involved in the stabilising loop that showed diurnal rhythmicity in its expression (*cipca*). The transcriptional regulation of these genes was measured in individuals from all emergence ranks (1–10). After the group emergence test, random elimination of two fish per group and overnight group housing separated by emergence order, triplicates of 3 × 8 fish (*n* = 72) were housed individually in separate containers (10 × 10 × 10 cm). Sampling occurred at six time points, i.e. in the middle of the light phase (7:00 hCT), 1 h before the dark phase (13:00 hCT), 1 h into the dark phase (15:00 hCT), in the middle of the dark phase (19:00 hCT), 1 h before the light phase (23:00 hCT) and 1 h into the light phase (1:00 hCT). Fish were sacrificed in ice water, and brains were dissected and preserved in RNALater (Ambion, Austin, TX). The bodies were preserved in liquid nitrogen for further analysis of hormones (see below). Triplicate samples were taken. The tissue was homogenised in 1 ml TRIzol reagent (Invitrogen), and total RNA was extracted according to the manufacturer’s instructions. cDNA synthesis was performed using the iScript kit (Bio-Rad Laboratories) according to the manufacturer’s instructions with forward and reverse primers for the aforementioned target genes and ippA (inducing PCN production A) as a reference (housekeeping) gene. Real-time quantitative PCR was performed using the Chromo4 Real-time PCR detection system (Bio-Rad Laboratories). All reactions were performed as technical triplicates. PCR analysis was performed using the following protocol: 95 °C 3 min, 40 cycles of 95 °C 15 s and 60 °C 45 s, and final melting curve of 81 cycles from 95 °C 1 min to 55 °C 10 s. Results were analysed using the ΔΔCt method. In short, ΔCt was calculated for each gene by subtracting the Ct values determined for the housekeeping gene from Ct values determined for the target genes. Then, the ΔCt values for EE were subtracted from the ΔCt values for LE fish. The values of the resulting ΔΔCt were then taken as the potentiator of 2 (2^(ΔΔCt)^), resulting in average fold change of emergence rank per time point.

Data analysis was performed using standard procedures [54]: A LOWESS curve (5-point window) was fitted over the data plotted over time and a sine wave was fitted subsequently over the resulting curve. The area under the curve (AUC) and the amplitude of the sine wave were further plotted against emergence rank (GraphPad Prism 6.0), and the correlation was analysed by a Spearman rank test (*N* = 72, significance accepted at *p* < 0.05).

### Determination of cortisol and melatonin levels

Whole body cortisol and melatonin concentrations were measured in the bodies of the fish used for the qPCR analysis of the brains. After weighing and pulverising them in liquid nitrogen, 500 μl ice-cold 1 × PBS buffer was added and the samples were vortexed for 1 min. Subsequently, 500 μl chloroform was added and vortexed for 1 min and then centrifuged at 1500*g* for 5 min. Following centrifugation, the organic layer of each sample containing melatonin and cortisol was transferred to a separate test tube. Extraction was repeated three times. Samples were kept overnight in the fume hood for evaporation of chloroform. The next day, 200 μl ice-cold 1 × PBS buffer was added, and 100 μl was used for the determination of melatonin, and 50 μl for cortisol, using ELISA kits (melatonin: Abelissa, Alachua, FL, 32615 USA; cortisol: Demetic Diagnostics GmbH, Kiel, Germany). Two-way ANOVA, with concentrations and time points as variables, indicated overall effects, and Sidaks’s multiple comparison test was used for the post hoc determination of differences between values per time point. Significance was accepted at *p* < 0.05.

A LOWESS curve (5-point window) was fitted over the data plotted over time, and a sine wave was fitted subsequently over the resulting curve. The AUC and the amplitude of the sine wave were further plotted against emergence rank (GrapPad Prism 6.0), and the correlation was analysed by a Spearman rank test (*N* = 72, significance accepted at *p* < 0.05).

### Activity patterns over diurnal/circadian cycle

In order to determine activity levels over diurnal and circadian cycles, individuals were automatically tracked over a period of 3 days under the usual light-dark regime (14:10 LD). Three rows of eight Plexiglas tanks (33 × 13 × 13 cm, capacity 3 l) were placed on a table surface (1.0 × 1.0 m). A flow through system (47.6 l h^−1^) provided each tank with fresh system water. Underneath, a retro-reflecting tape surface (1.2 × 1.2 m, Scotchlite 3 M, St. Paul, USA) reflected infrared light from three spots (U48R Univivi, Shenzhen, China), placed around an infrared firewire camera (Dragonfly, Point Grey Research Inc., Richmond, Canada) in a distance of 7 cm. The camera light unit was placed 1.7 m above the table.

Fish previously tested in the group emergence test in sets of ten, with random elimination of two, were placed individually in the tanks. Each row contained fish from the same group emergence test, i.e. 8 fish in three rows, resulting in 24 fish per experimental run, with three experimental runs (*N* = 3), resulting in *n* = 72. Food was provided ad libitum in the form of negatively buoyant pallets (Weekend, Tetra, USA). Fish were acclimated 30–48 h prior to recording. During the exposure to diurnal cycles (L:D), filming started at the middle of the light phase (7:00 hCT) at day 0 and lasted until 7:00 hCT of day 3 (L:D). The light was then kept on for another 3 days (72 h) in order to create constant light conditions (L:L), and filming continued until 7:00 hCT of day 3 L:L. Locomotion patterns were quantified as average velocity (*V*, mm s^−1^) over periods of 30 min, using EthoVision XT 6 (Noldus Information Technology b.v., Wageningen, The Netherlands).

For the analysis of the locomotion activity, a LOWESS curve (5-point window) was fitted over the activity data plotted over time and subsequently fitted sine wave yielded area under the curve (AUC, dimensionless) as an indicator for general locomotion activity and amplitude (mm s^−1^) as an indicator for activity fluctuation.

The rhythm strength of the 24-h component in activity data was gained as follows: after testing for multicollinearity in the explanatory variables by using variance inflation factors, a multivariate model predicting emergence rank was made, based on the remaining variables. Cook’s distance was calculated for all data points, but no outliers were found. Using likelihood ratio tests and Akaike’s information criterion, the optimal model was determined. The only explanatory variable of this optimal model was rhythm strength of the 24-h component. Visual inspection of the residuals revealed no violation by this model of statistical assumptions.

Finally, the maximum velocity (*V*_max_, mm s^−1^), i.e. the average of the maximum values for V during the first three 24-h periods (L:D), gave an indication for absolute swimming capacity. AUC, amplitude, rhythm strength and *V*_max_ were plotted against emergence rank (GraphPad Prism 6.0), and the correlation was analysed by a Spearman rank test (*N* = 72, significance accepted at *p* < 0.05).

## Additional files

Additional file 1: **Figure S1.** Evaluation of group emergence test by repeated single emergence. A) Dependence of single emergence time on the diurnal time point. Each individual fish was tested in a single emergence test at 1:00, 7:00 or 13:00 hCT on two subsequent days. This experiment was performed in a 3 × 3 design, so all possible combinations of two time points on consecutive days were tested. For each combination of times, 3 sets of 8 individuals were tested. The test was terminated after one hour. Emergence times of day 2 were plotted against emergence times of day 1. The results show a significant correlation (Spearman rank, *p* < 0.05) in all cases, indicating that the emergence time is not dependent on the time of day (log-log transformed scale). B) Single emergence times and group emergence ranks for light preference testing. Single emergence times of emergence from a darkened holding compartment to a darkened novel environment (log-transformed) were plotted over standard group emergence rank. The results show a significant correlation, suggesting that emergence, as a proxy for risk-taking, is independent of differences in light intensity between the two compartments (Spearman rank, *p* < 0.05). C) Single and group emergence times of all experiments. The results show a significant correlation, suggesting that emergence, as a proxy for risk-taking, is independent of social and environmental settings and consistent over time and across context (Spearman rank, significance accepted at *p* < 0.05, log-log transformed scale). (PDF 184 kb)

Additional file 2: **Figure S2.** No difference between performance of AB and TL fish in group emergence test. It was tested whether the observed behavioural variation in the performance in the group emergence test originated from differences between the original AB and TL lines, which had been used to generate the AB/TL line used in the present study. For this purpose, the emergence test was repeated with 6 groups of each 10 fish consisting of 5 AB and 5 TL fish in a ratio of 1:1. The results show no significant difference between the number of AB (black bars) and TL fish (grey bars) per emergence rank, indicating no difference between the two lines. Data analysis was performed using a Χ^2^ test (*N* = 6, significance accepted at *p* < 0.05). (PDF 34 kb)

Additional file 3: **Table S1.** Differently expressed genes in early emerging (EE) and late emerging (LE) fish. Analysis of the EE and LE transcriptome data show that 1478 genes out of 31,398 genes analysed were expressed significantly different (*p* < 0.05). Out of these genes, 43 are involved in the regulation of the biological clock (yellow). Genes upregulated in EE fish are marked red and genes upregulated in LE fish are marked blue. The table gives chromosome number, gene name, Ensembl gene-id, Entrez gene-id, ZFIN gene-id, GO accessions, Human homologues, fold change and *p*-value. (XLSX 317 kb)

Additional file 4: **Figure S3.** Correlation between expression patterns of clock-related genes and emergence rank. Area under the curve (AUC) and amplitude for relative expression patterns of *bmal1a* (A), *clock1a* (B), *per1a* (C), *cry1a* (D) and *cipca* (E) mRNA determined with quantitative real-time PCR (qPCR) in the brain tissue of fish from the entire range of emergence ranks (1–10). AUC values show a significant negative correlation with emergence rank for *bmal1a* and *cipca*, indicating decreasing expression activity with reduced risk-taking behaviour. Similarly, amplitude values (F–J) show a significant negative correlation with emergence rank for all genes, indicating increasingly dampened rhythmicity of the expression with reduced risk-taking behaviour. Solid lines indicate significant correlations (Spearman rank test, significance accepted at *p* < 0.05). (PDF 96 kb)

Additional file 5: **Figure S4.** Correlation between the concentration of cortisol and melatonin and emergence rank. The areas under the curve (AUC) and the amplitude were calculated for whole body cortisol and melatonin concentrations over time and plotted against emergence rank 1–10. The results show significant positive correlations of the AUC (A, B) and negative correlations for the amplitude (C, D) values for both, cortisol (A, B) and melatonin (C, D) (Spearman rank, significance accepted at *p* < 0.05), indicating increasingly dampened rhythmicity for the concentration of these hormones and reduced hormone production with reduced risk-taking behaviour. (PDF 63 kb)

Additional file 6: **Figure S5.** Correlation between diurnal pattern of locomotor activity and emergence rank under normal light/dark (LD) regime and under constant (LL) light regime. A) The strength of the diurnal rhythm (dimensionless) under LD plotted against emergence rank 1–10, showing a significant negative correlation. B) Amplitude of locomotion activity in units of swimming velocity (*V*, mm s^−1^) under LD plotted against emergence rank 1–10 showing a significant negative correlation. C) Area under the curve of locomotion activity (AUC, dimensionless) under LD plotted against emergence rank 1–10, showing a significant negative correlation. D) Maximum swimming velocity (*V*_max_, mm s^−1^) as average over three days under LD plotted against emergence rank 1–10 showing no significant correlation. E) Rhythm strength (dimensionless) under LL plotted against emergence rank 1–10, showing no significant correlation. F) Amplitude of locomotion activity in units of swimming velocity (*V*, mm s^−1^) under LL plotted against emergence rank 1–10 showing no significant correlation. G) Area under the curve of locomotion activity (AUC, dimensionless) under LL plotted against emergence rank 1–10 with no significant correlation (Spearman rank test, significance accepted at *p* < 0.05). The results indicate an increasingly dampened rhythmicity for locomotor activity under LD but less so under LL. A lack of this correlation in *V*_max_ indicates no difference in physiological capacity for locomotion. (PDF 130 kb)
