# Supplementary figures and images for: Biological clock function is linked to proactive and reactive personality types
Source: BMC Biol. 2018 Dec 21;16:148. doi: 10.1186/s12915-018-0618-0 (PMC6303931; doi:10.1186/s12915-018-0618-0)

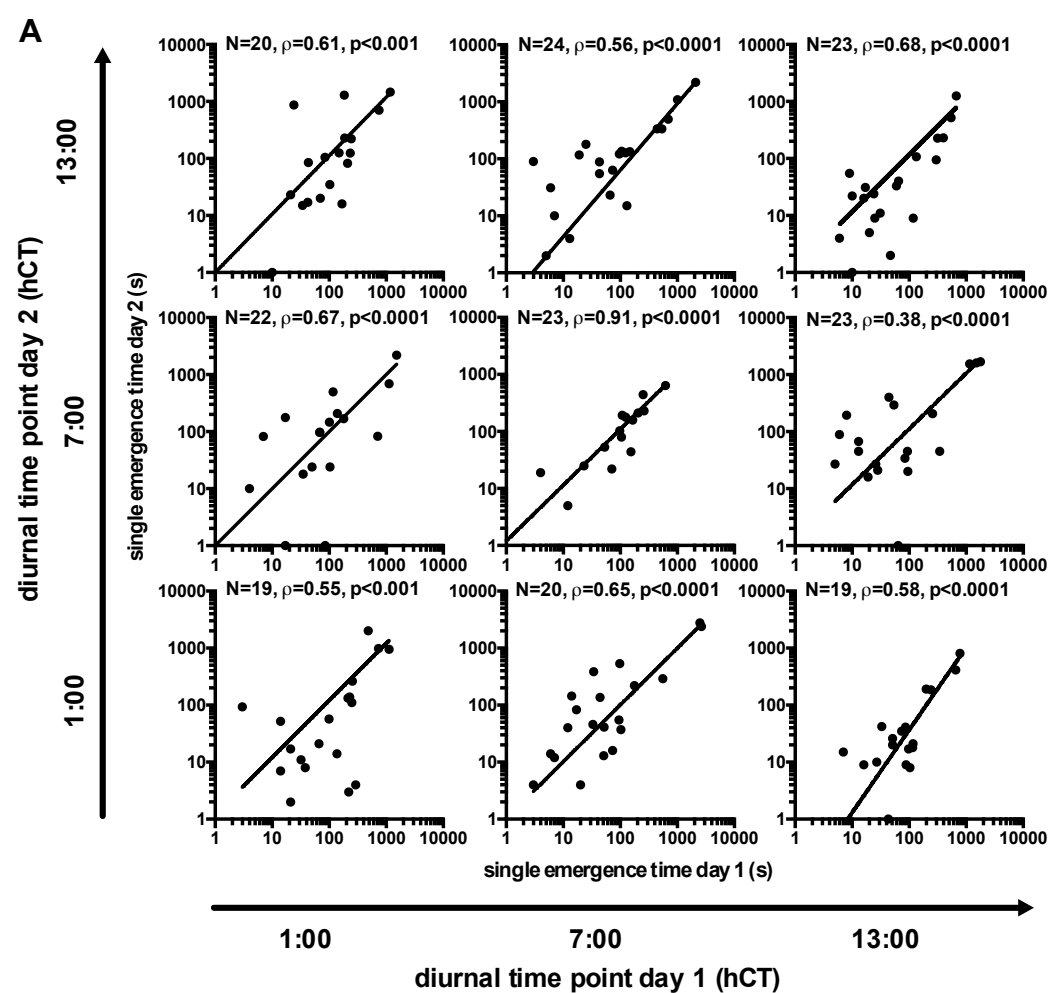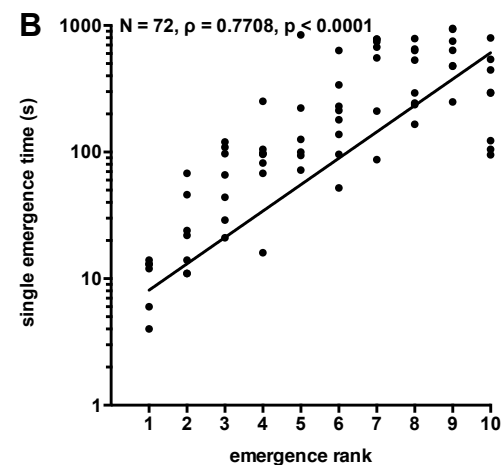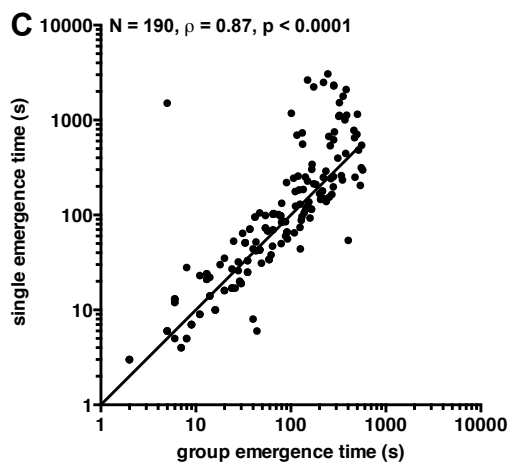

Supplement: Supplementary file 1 — Figure S1. Evaluation of group emergence test by repeated single emergence. A) Dependence of single emergence time on the diurnal time point. Each individual fish was tested in a single emergence test at 1:00, 7:00 or 13:00 hCT on two subsequent days. This experiment was performed in a 3 × 3 design, so all possible combinations of two time points on consecutive days were tested. For each combination of times, 3 sets of 8 individuals were tested. The test was terminated after one hour. Emergence times of day 2 were plotted against emergence times of day 1. The results show a significant correlation (Spearman rank, p < 0.05) in all cases, indicating that the emergence time is not dependent on the time of day (log-log transformed scale). B) Single emergence times and group emergence ranks for light preference testing. Single emergence times of emergence from a darkened holding compartment to a darkened novel environment (log-transformed) were plotted over standard group emergence rank. The results show a significant correlation, suggesting that emergence, as a proxy for risk-taking, is independent of differences in light intensity between the two compartments (Spearman rank, p < 0.05). C) Single and group emergence times of all experiments. The results show a significant correlation, suggesting that emergence, as a proxy for risk-taking, is independent of social and environmental settings and consistent over time and across context (Spearman rank, significance accepted at p < 0.05, log-log transformed scale). (PDF 184 kb) [file 12915_2018_618_MOESM1_ESM.pdf]

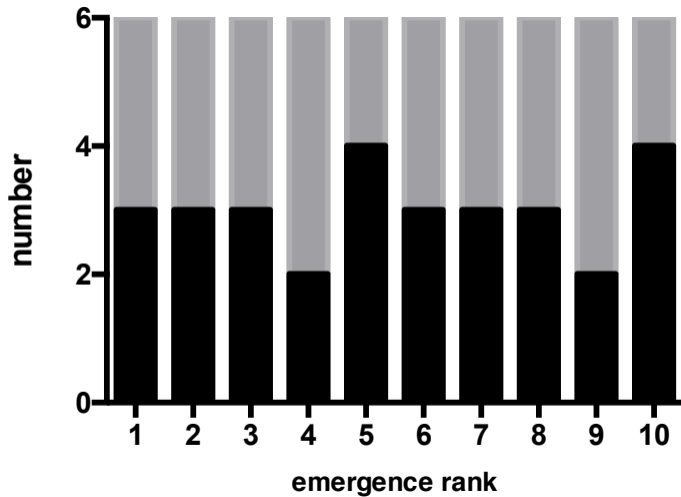

Supplement: Supplementary file 2 — Figure S2. No difference between performance of AB and TL fish in group emergence test. It was tested whether the observed behavioural variation in the performance in the group emergence test originated from differences between the original AB and TL lines, which had been used to generate the AB/TL line used in the present study. For this purpose, the emergence test was repeated with 6 groups of each 10 fish consisting of 5 AB and 5 TL fish in a ratio of 1:1. The results show no significant difference between the number of AB (black bars) and TL fish (grey bars) per emergence rank, indicating no difference between the two lines. Data analysis was performed using a Χ2 test (N = 6, significance accepted at p < 0.05). (PDF 34 kb) [file 12915_2018_618_MOESM2_ESM.pdf]

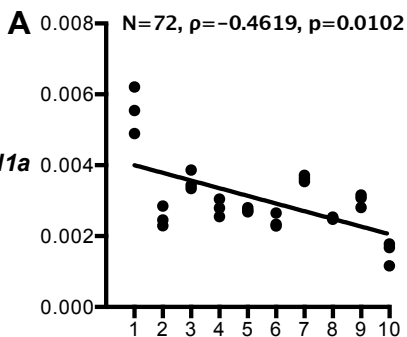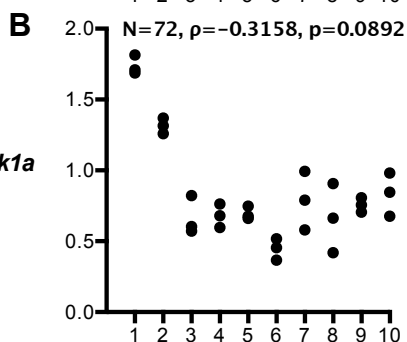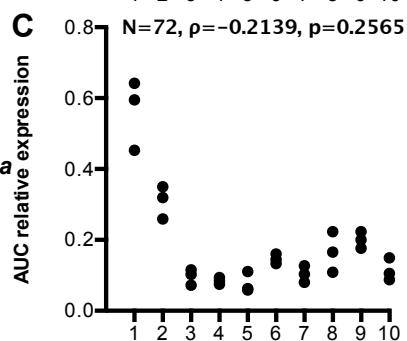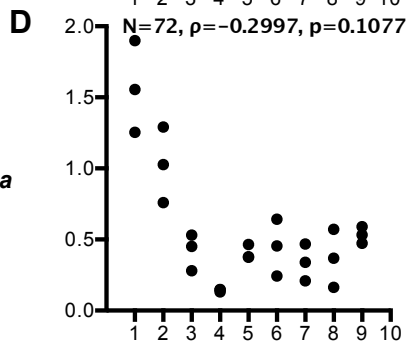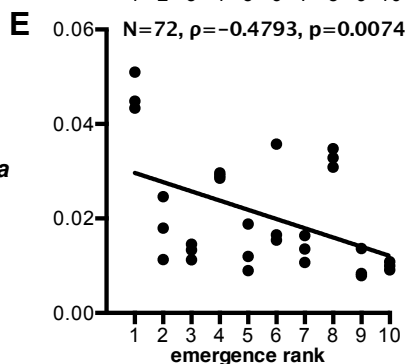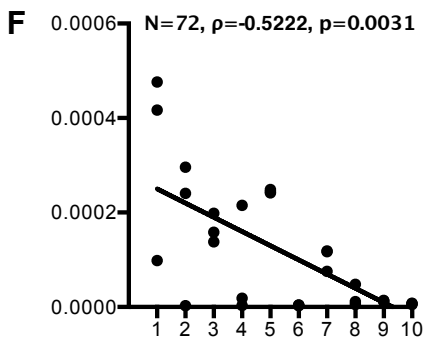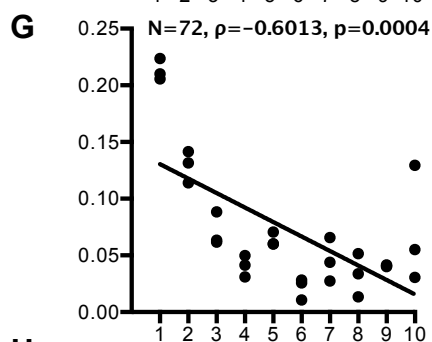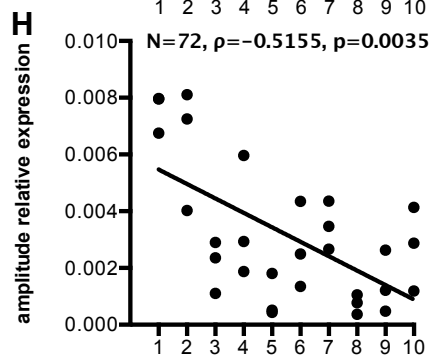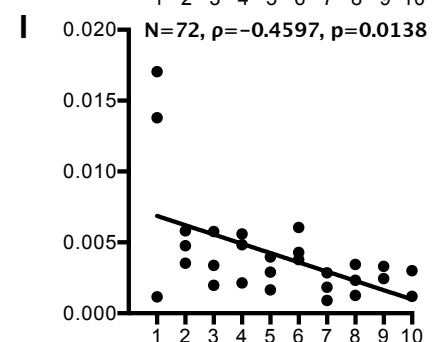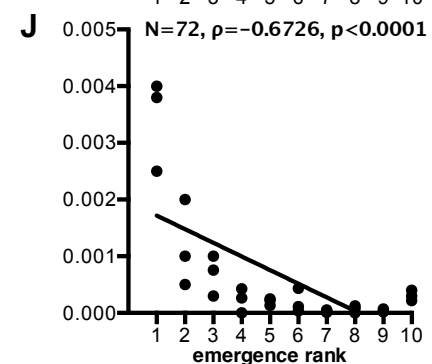

Supplement: Supplementary file 4 — Figure S3. Correlation between expression patterns of clock-related genes and emergence rank. Area under the curve (AUC) and amplitude for relative expression patterns of bmal1a (A), clock1a (B), per1a (C), cry1a (D) and cipca (E) mRNA determined with quantitative real-time PCR (qPCR) in the brain tissue of fish from the entire range of emergence ranks (1–10). AUC values show a significant negative correlation with emergence rank for bmal1a and cipca, indicating decreasing expression activity with reduced risk-taking behaviour. Similarly, amplitude values (F–J) show a significant negative correlation with emergence rank for all genes, indicating increasingly dampened rhythmicity of the expression with reduced risk-taking behaviour. Solid lines indicate significant correlations (Spearman rank test, significance accepted at p < 0.05). (PDF 96 kb) [file 12915_2018_618_MOESM4_ESM.pdf]

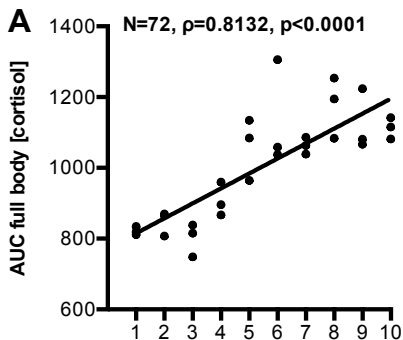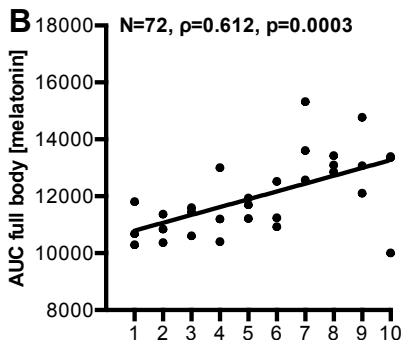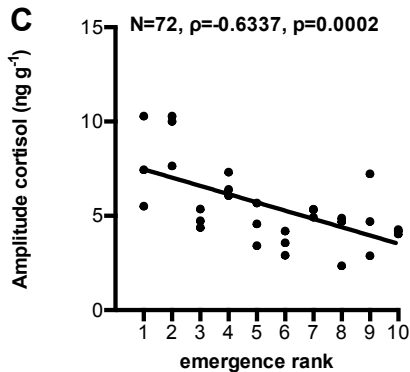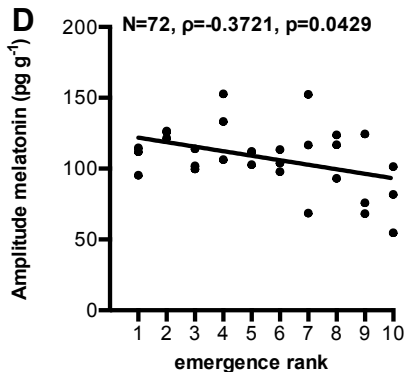

Supplement: Supplementary file 5 — Figure S4. Correlation between the concentration of cortisol and melatonin and emergence rank. The areas under the curve (AUC) and the amplitude were calculated for whole body cortisol and melatonin concentrations over time and plotted against emergence rank 1–10. The results show significant positive correlations of the AUC (A, B) and negative correlations for the amplitude (C, D) values for both, cortisol (A, B) and melatonin (C, D) (Spearman rank, significance accepted at p < 0.05), indicating increasingly dampened rhythmicity for the concentration of these hormones and reduced hormone production with reduced risk-taking behaviour. (PDF 63 kb) [file 12915_2018_618_MOESM5_ESM.pdf]

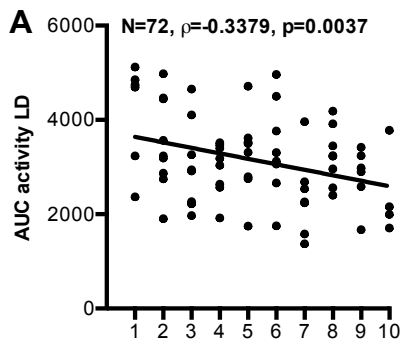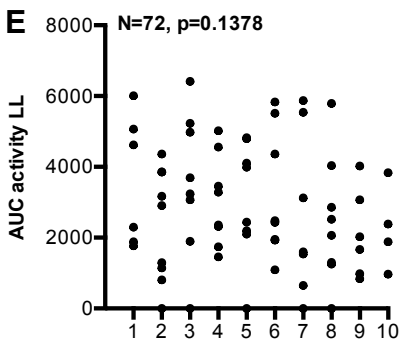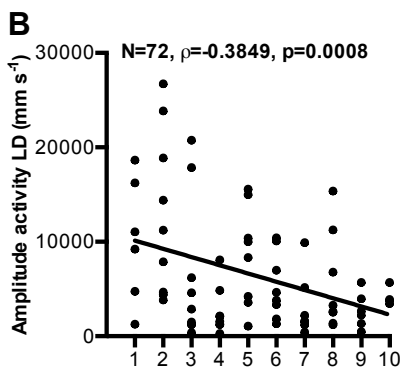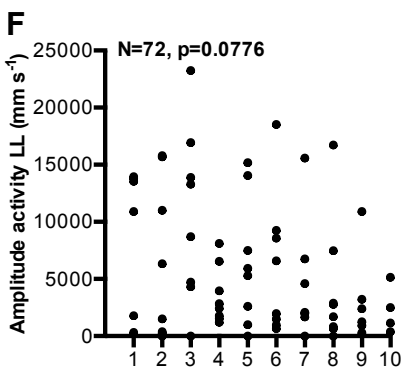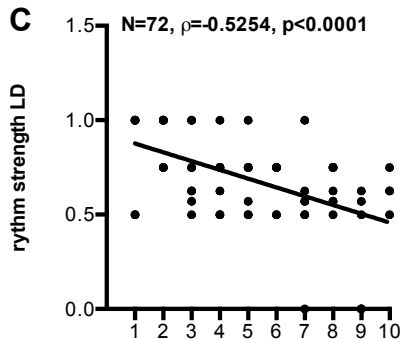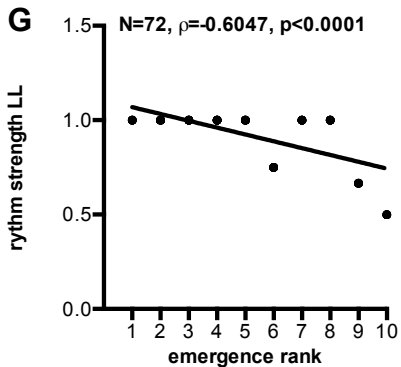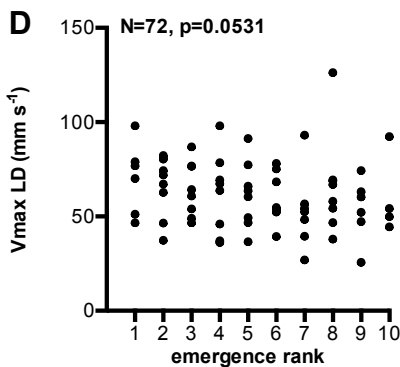

Supplement: Supplementary file 6 — Figure S5. Correlation between diurnal pattern of locomotor activity and emergence rank under normal light/dark (LD) regime and under constant (LL) light regime. A) The strength of the diurnal rhythm (dimensionless) under LD plotted against emergence rank 1–10, showing a significant negative correlation. B) Amplitude of locomotion activity in units of swimming velocity (V, mm s−1) under LD plotted against emergence rank 1–10 showing a significant negative correlation. C) Area under the curve of locomotion activity (AUC, dimensionless) under LD plotted against emergence rank 1–10, showing a significant negative correlation. D) Maximum swimming velocity (Vmax, mm s−1) as average over three days under LD plotted against emergence rank 1–10 showing no significant correlation. E) Rhythm strength (dimensionless) under LL plotted against emergence rank 1–10, showing no significant correlation. F) Amplitude of locomotion activity in units of swimming velocity (V, mm s−1) under LL plotted against emergence rank 1–10 showing no significant correlation. G) Area under the curve of locomotion activity (AUC, dimensionless) under LL plotted against emergence rank 1–10 with no significant correlation (Spearman rank test, significance accepted at p < 0.05). The results indicate an increasingly dampened rhythmicity for locomotor activity under LD but less so under LL. A lack of this correlation in Vmax indicates no difference in physiological capacity for locomotion. (PDF 130 kb) [file 12915_2018_618_MOESM6_ESM.pdf]
